# Burden and impact of arrhythmias in asthma‐related hospitalizations: Insight from the national inpatient sample

**DOI:** 10.1002/joa3.12452

**Published:** 2020-11-17

**Authors:** Muhanad Taha, Tushar Mishra, Mohamed Shokr, Aditi Sharma, Mazen Taha, Lobelia Samavati

**Affiliations:** ^1^ Department of Internal Medicine Detroit Medical Centre/Wayne State University Detroit MI USA; ^2^ Cardiology Department Leon H. Charney Division of Cardiology, Cardiac Electrophysiology NYU Langone Health New York University Grossman School of Medicine New York NY USA; ^3^ Faculty of Medicine Cairo University Giza Egypt; ^4^ Department of Pulmonary Critical Care and Sleep Division Wayne State University Detroit MI USA

**Keywords:** asthma disease, asthma exacerbation, atrial fibrillation, cardiac arrhythmia, healthcare burden, in‐hospital mortality

## Abstract

**Background:**

This study aimed to analyze the burden and impact of cardiac arrhythmias in adult patients hospitalized with asthma exacerbation using the nationwide inpatient database.

**Methods:**

We used the National Inpatient Sample (NIS) database (2010‐2014) to identify arrhythmias in asthma‐related hospitalization and its impact on inpatient mortality, hospital length of stay (LOS), and hospitalization charges. We also used multivariable analysis to identify predictors of in‐hospital arrhythmia and mortality.

**Results:**

We identified 12,988,129 patients hospitalized with primary diagnosis of asthma; among them, 2,014,459(16%) patients had cardiac arrhythmia. The most frequent arrhythmia identified is atrial fibrillation (AFib) (8.95%). The AFib and non‐AFib arrhythmia group had higher mortality (3.40% & 2.22% vs 0.74%), mean length of stay (LOS) (5.9 & 5.4 vs 4.2 days), and hospital charges ($53,172 & $51,105 vs $34,585) as compared to the non‐arrhythmia group (*P* < .005). Predictors of arrhythmia in asthma‐related hospitalization were history of PCI or CABG, valvular heart disease, congestive heart failure (CHF), and acute respiratory failure. Predictors of higher mortality in arrhythmia group were acute respiratory failure, sepsis, and acute myocardial infarction.

**Conclusions:**

Around 16% of adult patients hospitalized with asthma exacerbation experience arrythmia (mostly AFib 8.95%). The presence of arrhythmias was associated with higher in‐hospital mortality, LOS, and hospital charges in hospitalized asthmatics.

## Graphical abstract

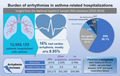

## 1 INTRODUCTION

Asthma is the most common lung disease that is increasingly recognized in adults. Asthma affects around 19.2 million adults in the United States.^1^ Around 80% of asthma population is above 18 years old and 14% is above 65 years old.^1^ In 2016, 1.2 million emergency room visits, and 7.3 million office visits were asthma related, placing a huge burden on the healthcare system.^2^

Cardiovascular disease is the most frequent cause of death among hospitalized asthma patients.^3^ Several studies suggested that asthma is an independent risk factor for cardiovascular disease (CVD)^4^, ^5^ and arrhythmias.^6^, ^7^, ^8^, ^9^ Previous studies described high prevalence of tachycardia, premature ventricular contractions^6^ and AFib^7^, ^8^, ^9^ among patients with asthma. The exact mechanism of arrythmia in asthma disease is poorly understood and likely multifactorial. The pathogenesis of asthma is characterized by chronic inflammation of airways.^10^, ^11^. This inflammation may play a role in developing arrythmias in asthmatics as inflammation is a well‐established risk factor for arrhythmias.^12^ Other than inflammation, respiratory failure and bronchodilator therapy are also possible mechanisms of arrythmias in asthma disease.

There is limited data on the burden and impact of arrythmia in asthma hospitalizations in a population‐based study. Therefore, we conducted this study to examine the nationwide type and frequency of arrhythmia in asthma‐related hospitalizations and its impact on mortality, length of stay, and hospital charges. We also identified significant predictors for arrhythmia and mortality in asthma hospitalizations.

## 2 METHODS

### 2.1 Data source

For our analysis, we used the National Inpatient Sample (NIS) database over a 4‐year period between January 1, 2010 and December 31, 2014. The NIS is the largest publicly available all‐payers' data set in the United States sponsored by the Agency for Health Care Quality and Research (AHRQ).NIS contains inpatient admissions and discharge data from about 1050 US hospitals across 45 states with approximately 7 million unweighted discharges each year. The NIS contains data for each hospital stay including patient demographics, total charges, length of stay, admission, and discharge status as well as primary and secondary diagnoses using the international classification of diseases, ninth edition (ICD‐9) codes. More details on the database are available on the HCUP website.^13^

### 2.2 Study population

Adult patients (>18 years old) with Asthma‐related hospitalizations and arrhythmia were identified using ICD‐9‐CM (International Classification of Diseases, Ninth Revision, and Clinical Modification) code 128 and 106, respectively. The comorbidities and subtypes of arrhythmias were also identified using the ICD‐9‐CM codes. To include only asthma patients, we excluded patients with secondary diagnosis of COPD.

### 2.3 Study outcomes

We used the hospital length of stay (LOS), hospital charges and in‐hospital mortality to assess the impact of arrhythmia on asthma‐related hospitalization outcomes. We assessed and compared the baseline demographics, hospital‐level characteristics, and associated comorbidities in asthma hospitalizations with atrial fibrillation (AFib) and non‐AFib arrhythmia vs no arrhythmia. We also assessed the impact of multiple comorbidities on arrhythmia incidence and subsequent inpatient mortality during asthma hospitalizations.

### 2.4 Statistical analyses

National estimates were produced using previously described methodology by the HCUP.^13^ After applying appropriate weights, we compared various baseline characteristics (eg, patients ‘demographics, comorbidities, and hospital outcomes) between the study groups (AFib and non‐AFib arrhythmia group vs non‐arrhythmia group). Pearson chi‐square test was used to compare categorical variables, while linear combination tests were used to compare continuous variables. The results were reported as percentage and mean ± standard deviation, respectively. We then built multivariate logistic regression model using backward selection method to evaluate predictors of arrhythmia in asthma patients and in hospital mortality in asthma patients with arrhythmia. All relevant demographic characteristics, hospital characteristics, and comorbidities were evaluated with univariate analysis first, and the clinically relevant variables were used for multivariate analysis. The results were reported as adjusted odds ratio, 95% confidence intervals and p‐values. This analysis was done using STATA software version 15.1 (Stata Corporation, College Station, TX, USA).

## 3 RESULTS

### 3.1 Burden of arrhythmias

**FIGURE 1:**
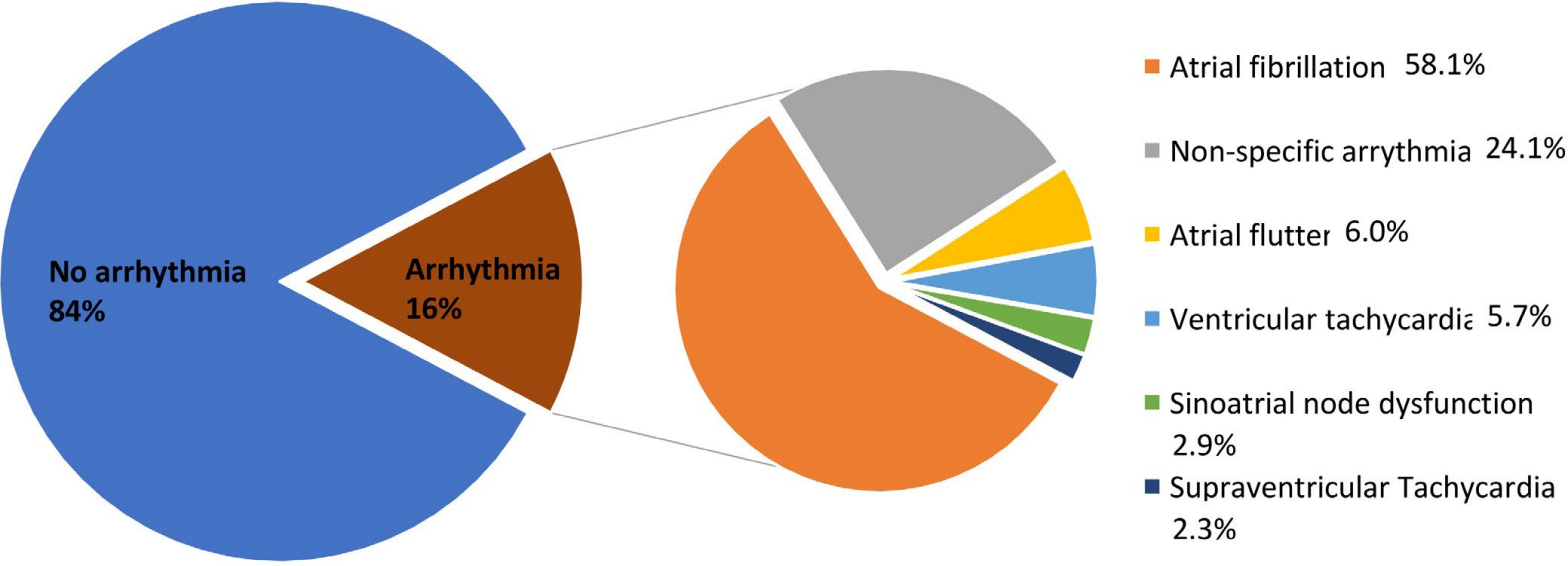
The most frequent arrhythmia in asthma hospitalization

**FIGURE 2:**
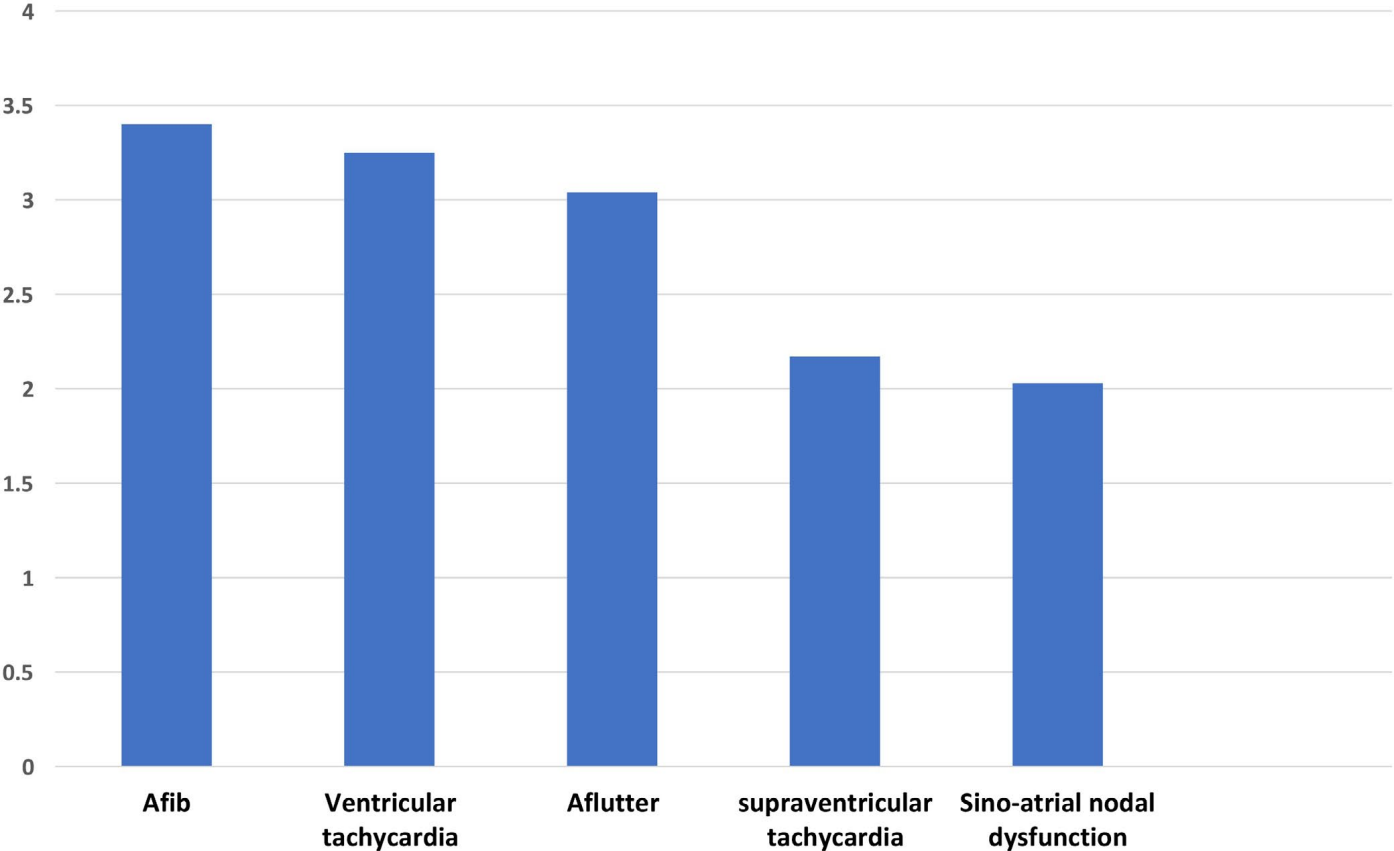
In‐hospital mortality in asthma hospitalizations per arrhythmia type

We identified a total of 12 988 129 patients admitted with primary diagnosis of asthma disease from 2010 to 2014. Among those, cardiac arrhythmia was reported in 2 014 459 (16%) patients. The most frequent arrhythmias in the descending frequency were atrial fibrillation (8.95%), followed by non‐AFib arrhythmia including: nonspecific arrhythmia (3.86%), atrial flutter (0.93%),ventricular tachycardia (0.88%), sinoatrial nodal dysfunction (0.44%), and paroxysmal supraventricular tachycardia(0.35%)(Figure 1). In‐hospital mortality rate in asthma hospitalizations was higher in patients with atrial fibrillation (3.40%) followed by ventricular tachycardia (3.25%). (Figure 2) showed mortality rates of other types of arrhythmia. The nonspecific arrythmia has ICD‐9 code 427.9, and this code was used to report arrhythmia when no further specificity was available.

### 3.2 Baseline characteristics

Asthma patients with AFib were predominantly older {mean age ~72 (AFib) vs 52 (non‐AFib)and 46 years (no arrhythmia)}, white {75.8% (AFib) vs 59.9% (non‐AFib) and 58.8% (no arrhythmia)}, and Medicare beneficiaries {76.8% (AFib) vs 42.5% (non‐AFib) and 34.0% (no arrhythmia)} as compared to those with non‐AFib arrhythmia and those without arrhythmia(*P* < .005) (Table 1).

**TABLE 1:** Baseline characteristics of asthma patients with AFib arrhythmia vs Non‐AFib arrhythmia vs No arrhythmia

Variable	No arrhythmia	AFib arrhythmia	Non‐AFib arrhythmia	*P*‐value
Mean age	46.13 ± 22.88	72.24 ± 12.91	52.18 ± 22.55	<.005
Female	68.08%	63.58%	65.37%	<.005
Race				
White	58.80%	75.76%	59.9%	<.005
Black	23.53%	12.47%	25.14%	
Hispanic	11.78%	6.61%	9.82%	
Asian or Pacific Islander	1.90%	2.44%	2.21%	
Native American	0.72%		0.58%	
Other	3.28%	2.29%	3.19%	
Primary expected payer				
Medicare	33.97%	76.82%	42.49%	<.005
Medicaid	27.16%	6.21%	21.71%	
Private Insurance	29.93%	14.03	26.81%	
Self‐pay	5.13	1.42%	5.50%	
Comorbidities				
Hypertension	44.19%	73.59%	54.37%	<.005
CHF	6.25%	27.12%	9.88%	<.005
Acute Myocardial Infarction	1.37%	3.87%	3.44%	<.005
Peripheral vascular disorders	3.16%	9.97%	5.10%	<.005
Valvular disorders	2.38%	10.90%	4.49%	<.005
Venous Thromboembolism	5.30%	9.22%	6.09%	<.005
Solid tumor without metastasis	1.18%	2.06%	1.50%	<.005
Lymphoma	0.48%	1.01%	0.67%	<.005
Diabetes with chronic complications	3.80%	7.37%	4.60%	<.005
Obesity	18.82%	22.80%	21.17%	<.005
Renal failure	7.07%	23.01%	10.36%	<.005
Liver disease	2.96%	2.56%	3.14%	<.005
Pulmonary Circulation disorders	1.67%	6.58%	3.25%	<.005

Percentage of female patients were higher in all the study groups (63.6% for AFib, 65.4% for non‐AFib, and 68.1% for non‐arrhythmia groups, *P* < .005) (Table 1). We analyzed the prevalence of comorbidities among hospitalized asthmatics with and without arrhythmia. The AFib and non‐AFib arrhythmia groups were more likely to have cardiac comorbidities such as CHF{27.1% (AFib), 9.90% (non‐AFib) and 6.25% (no arrhythmia)}, hypertension {73.6% (AFib), 53.4% (non‐AFib), and 44.2% (no arrhythmia)}, and valvular heart diseases {10.9% (AFib), 4.49% (non‐AFib), and 2.38% (no arrhythmia)} as compared to non‐arrhythmia group (*P* < .005). Comparison of other comorbidities is also shown in Table 1.

### 3.3 Impact of arrhythmia on mortality and outcomes of asthma hospitalizations

Patients developed arrhythmia during asthma hospitalization were associated with higher in‐hospital mortality rate (3.40% & 2.22% for AFib and non‐AFib arrhythmia groups, respectively, vs 0.74% for non‐arrhythmia group, *P* < .005). Similarly, the mean length of stay (days) (5.88 &5.40 vs 4.24, *P* < .005), total hospital charges ($53,172 & $51,105 vs $34,585, *P* < .005), and transfer rate (24.6% & 12.8% vs 9.8%) were significantly higher in the AFib and non‐AFib arrhythmia groups as compared to the non‐arrhythmia group (*P* < .005) (Table 2).

**TABLE 2:** Impact of AFib and Non‐AFib arrhythmia on mortality and outcomes of asthma hospitalizations

Variable	No arrhythmia	AFib arrhythmia	Non‐AFib arrhythmia	*P*‐value
All cause in hospital mortality	0.74%	3.40%	2.22%	<.005
Disposition of Patient				
Routine	75.52%	48.69%	68.12%	<.005
Transfer to short‐term Hospital	1.49%	2.34%	2.50%	<.005
Other Transfers (nursing facility, intermediate care facility, Another Facility)	9.75%	24.63%	12.81%	<.005
Home Health Care	10.86%	20.27%	12.77%	<.005
Against Medical Advice	1.55%	0.58%	1.48%	<.005
Length of Stay (days) Mean ± SD	4.24 ± 5.44	5.88 ± 6.35	5.40 ± 6.87	<.005
Total hospital charges (Mean)	$34 585 ± 52 038	$53 172 ± 77 285	$51 105 ± 80 152	<.005

### 3.4 Predictors of arrhythmia in asthma‐related hospitalization

**FIGURE 3:**
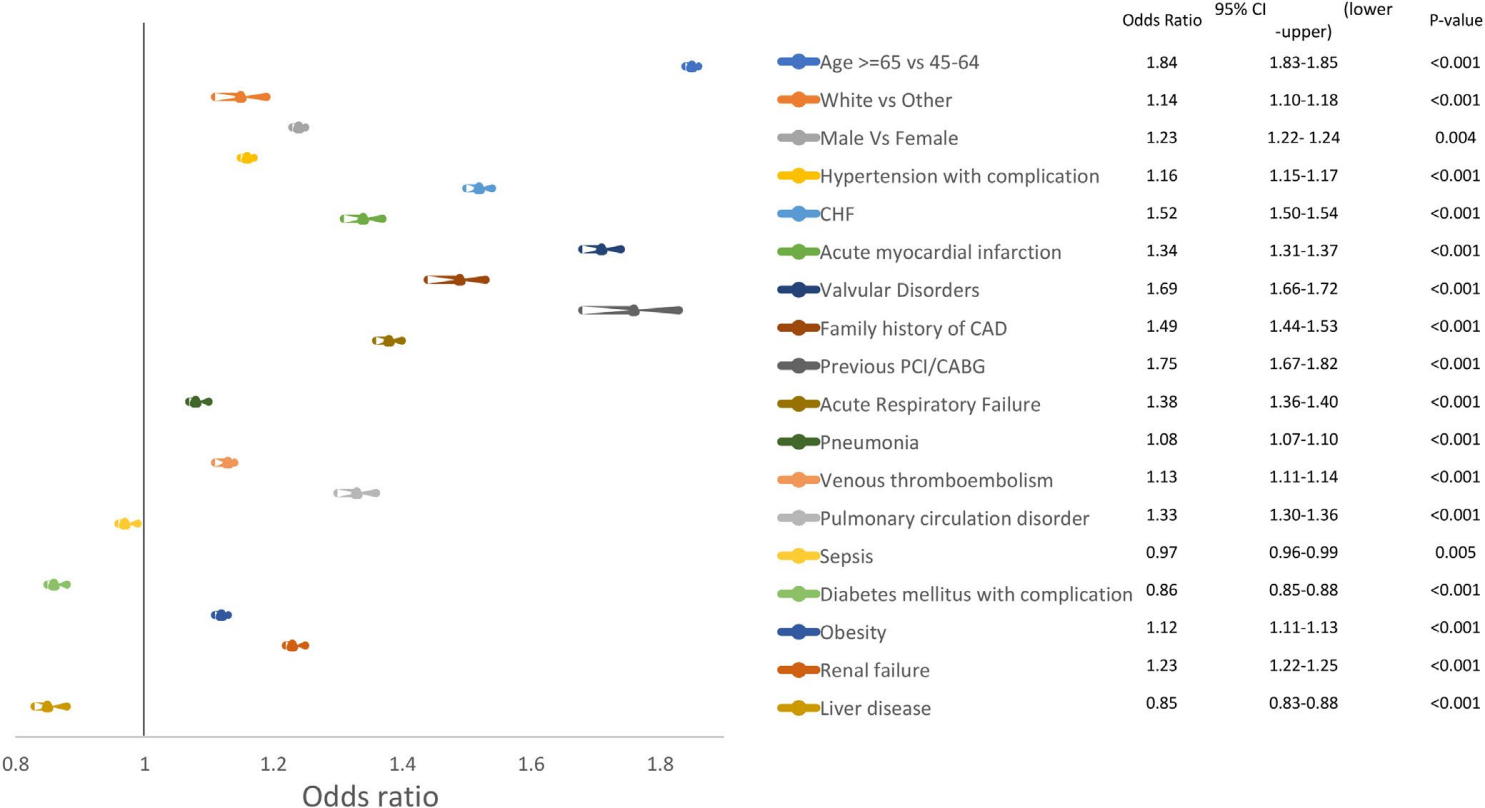
Multivariable predictors of arrhythmia among asthma hospitalization

We found that advanced age (OR 1.84: >65 vs 45‐64 years, CI 1.83‐1.85, *P* < .001), male sex (OR 1.23, CI 1.22‐1.24, P 0.004), and white race (OR 1.14, CI 1.10‐1.18, *P* < .001) had significant higher odds of arrhythmia as displayed in Figure 3. Higher odds of arrhythmia were associated with comorbid conditions such as history of PCI/CABG (OR 1.75,CI 1.67‐1.82, *P* < .001), valvular heart disease (OR 1.69, CI 1.66‐1.72, *P* < .001), CHF(OR 1.52, CI 1.50‐1.54, *P* < .001), family history of CAD (OR 1.49, CI 1.44‐1.53, *P* < .001), acute respiratory failure (OR 1.38, CI 1.36‐1.40, *P* < .001), and pulmonary circulation disorders (OR 1.33, CI 1.30‐1.36, *P* < .001) (Figure 3).

### 3.5 Predictors of mortality in asthma‐related hospitalizations with arrhythmia

**FIGURE 4:**
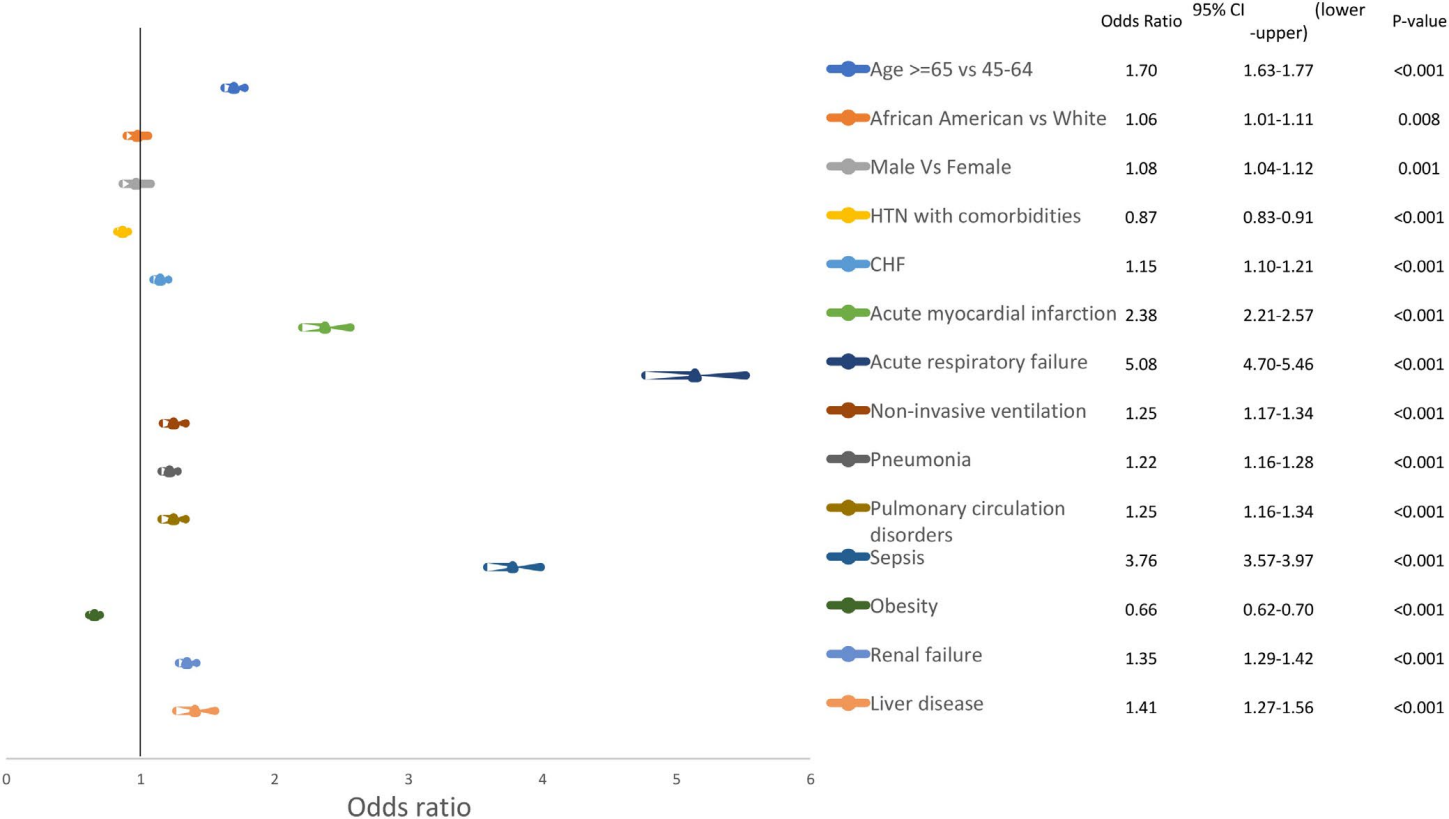
Multivariable predictors of mortality among asthma hospitalization with arrhythmia

We found that age group > 65, male, and white gender predict higher inpatient mortality in the arrhythmia group. As shown in Figure 4, multiple comorbidities were independent predictors of in‐hospital mortality in the arrhythmia group such as acute respiratory failure (OR 5.08, CI 4.70‐5.46, *P* < .001), sepsis (OR 3.76, CI 3.57‐3.97, *P* < .001), acute myocardial infarction (OR 2.38, CI 2.21‐2.57, *P* < .001), renal Failure (OR 1.35, CI 1.29‐1.42, *P* < .001), pneumonia (OR 1.22, CI 1.16‐1.28, *P* < .001), and CHF (OR 1.15, CI 1.10‐1.21, *P* < .001).

## 4 DISCUSSION

The important findings of this study are as follows: (a)one out of six patients (16%)hospitalized for asthma exacerbation experienced in‐hospital arrhythmia and AFib was the most common arrhythmia (Figure 1).(B) Cardiac arrythmias complicating asthma hospitalizations were associated with higher in‐hospital mortality, length of stay, and hospital charges (Table 2). (c) Predictors of arrhythmia in asthma‐related hospitalization were history of PCI or CABG, valvular heart disease, congestive heart failure (CHF), and acute respiratory failure (Figure 3). Predictors of higher mortality in arrhythmia group were acute respiratory failure, sepsis, and acute myocardial infarction (Figure 4). To our knowledge, this is the largest population‐based study to systemically report the burden and impact of arrhythmias in asthma‐related hospitalizations.

Asthma has been shown by several studies to be associated with arrhythmias. Warnier^6^ showed high prevalence of tachycardia and premature ventricular tachycardia in asthmatics while Cepelis^7^ and Tattersall^8^ showed high prevalence of AFib. However, our study is the only study to examine the frequency of other types of arrhythmias (like atrial flutter, ventricular tachycardia, etc). The frequency of AFib was about 8.95% in our study, which was higher than the frequency observed in Warnier,^6^ Cepelis,^7^ and Tattersall^8^ (0.6%, 4.5%–6%, and 1.3% respectively). The main reason is that these studies followed up asthma patients in clinic who generally had mild disease compared to our hospitalized patients and arrhythmias have been found to be associated with disease severity.^7^, ^14^ However, Carter^15^ conducted a large study examining the prevalence of AFib in 60 424 hospitalized asthmatics and found lower prevalence than our study (4.1% vs 8.95%). The cause of this difference is unclear, but it may be because of low cardiac comorbidities reported in Carter P’s study cohort compared to our cohort. Also, our study may overestimate the prevalence of AFib by not recognizing patients with recurrent admissions.

This study indicates that arrhythmia group presented with significantly higher in‐hospital mortality, length of stay and hospitalization charges. To our knowledge, no previous studies examined the impact of arrhythmia in asthma hospitalization. The overall in‐hospital mortality in asthma‐related hospitalizations was 0.8% and as high as 9.8% in patients requiring mechanical ventilation/intubation.^16^, ^17^ We found the in‐hospital mortality of 0.74% with no arrhythmia, 2.22% with non‐AFib arrhythmias, and 3.40% in AFib arrhythmia.

The association between asthma and AFib is not fully understood but might be explained by the following mechanisms. The first mechanism is related to respiratory failure associated with asthma exacerbation. We observe a higher risk of arrhythmia and mortality among asthma patients with respiratory failure requiring invasive or noninvasive ventilation (Figures 3 and 4). This finding is similar to previous studies on patients with asthma as well as patients with COPD.^18^, ^19^ Hypoxemia, hypercapnia and both respiratory and metabolic alkalosis developed in respiratory failure might contribute to the higher risk of arrhythmia among these patients.^20^, ^21^, ^22^, ^23^

The second mechanism is based on the association between inflammation and arrhythmias. Chronic airway inflammation is the pathogenesis of asthma disease. Inflammatory cells accumulate in the airway, activate cytokines, and enhance airway remodeling.^10^, ^11^ Previous studies demonstrated that asthma is not just a local inflammatory disease but rather a systemic inflammatory disease with high serum levels of inflammatory markers.^24^, ^25^ This systemic inflammation can increase the risk of arrhythmias directly^26^, ^27^ or indirectly by enhancing the formation and rupture of coronary atherosclerotic plaques.^12^ Tattersall^8^ demonstrated that IL‐6, D‐dimer, and TNF‐α R1 were independent risk factors for Afib among asthmatics. Asthma association with higher risk of CAD as previously mentioned^4^, ^5^ and this further increase the risk of arrhythmias.

The third mechanism is related to asthma therapy. Several studies provided evidence that asthma therapy like bronchodilators and corticosteroids increased the risk of arrhythmias^9^, ^28^, ^29^ and this might confound the association between asthma and arrhythmia. Unfortunately, our data does not contain information about medications used during hospitalization. However, Chan^9^ study demonstrated that asthma patients were at higher risk of new onset AFib independent of corticosteroid and bronchodilators use.

The association between cardiac comorbidities and arrhythmia is solid and widely cited. A similar association was noticed in our study with increased risk of arrhythmias especially AFib in patients with cardiac comorbidities (Figure 3). Some cardiac comorbidities were also independent predictors of mortality like myocardial infarction, congested heart failure, cardiogenic shock, and history of cardiac arrest (Figure 4).

Renal failure is independently associated with higher risk of arrhythmia. This is consistent with numerous studies which showed that patients with renal failure have high prevalence of cardiac arrhythmias and sudden cardiac death.^30^

It is important to mention that despite Whites predominate the AFib group, it does not mean that whites are at higher risk of AFib. Further analysis examining the probability of AFib in each race group in needed.

## 5 LIMITATIONS

This study has few limitations. First, the NIS database has the possibility of coding errors as it is an administrative dataset. It is possible that medical coders mistake asthma for COPD especially in elderly population. This might overestimate risk of arrhythmias in our study as COPD has higher prevalence of arrhythmias compared to asthmatics.^31^ Second, information about medications used for treatment, like bronchodilators and corticosteroids which might confound the association between arrhythmia and asthma, is not typically available in the NIS database. Our study, like any other observational studies, is prone to bias as a result of unmeasured confounders. For example, we found that arrhythmia is less likely to occur in asthmatics with liver disease, sepsis, and diabetes mellitus. This finding is likely affected by confounders and even if it is statistically significant, it is less likely to be clinically significant. Third, this study is limited to inpatient outcomes only as outpatient data is not available. Also, data about severity of asthma and long‐term incidence and impact of arrhythmia are also not available. Fourth, being unable to recognize readmissions might overestimate the prevalence of arrhythmia among asthma hospitalizations. Despite the limitations previously mentioned, the bias is likely minimal because of the large sample size available in the NIS database.

## 6 CONCLUSION

By utilizing the large data from NIS cohorts, we identified that cardiac arrhythmias are highly prevalent in asthma related‐hospitalizations. The presence of cardiac arrhythmia, including A fib and non‐A fib in asthmatics was associated significantly with higher all‐cause mortality, length of stay and hospital charges. Further studies needed to identify the reasons for increased risk of arrhythmia in asthma population.

## CONFLICT OF INTEREST

The authors have no conflict of interest.
